# Antibiotic Use in Veterinary

**DOI:** 10.3390/antibiotics11111539

**Published:** 2022-11-03

**Authors:** Manuel San Andres

**Affiliations:** Department of Pharmacology and Toxicology, Faculty of Veterinary Medicine, Universidad Complutense de Madrid, 28040 Madrid, Spain; misanand@vet.ucm.es

Since the introduction of antibiotics in the 1930s, the form and philosophy of their use has changed considerably. After an initial phase of great hope, a new front for the control and even the eradication of infectious diseases was opening up. In fact, one of the most infamous quotes in the history of biomedicine is: “*It is time to close the book on infectious diseases, and declare the war against pestilence won*.” This was long attributed to the United States Surgeon General, Dr. William H. Stewart (1965–1969); however, the primary source for the quote has never been identified. In recent years, that euphoria has been met with a harsh reality: a "gap" in the discovery of new antibiotics since 1990 and the exponential growth of resistance.

At this time, there are many agencies and public and private organizations that alert and offer recommendations for dealing with the problem of antibiotic resistance. Specifically, the European Union, through the REGULATION (EU) 2019/6 OF THE EUROPEAN PARLIAMENT AND OF THE COUNCIL, states that *Antimicrobial resistance to medicinal products for human use and veterinary medicinal products is a growing health problem in the Union and worldwide. Due to the complexity of the problem, its cross-border dimension and the high economic burden, its impact goes beyond its severe consequences for human and animal health and has become a global public health concern that affects the whole of society and requires urgent and coordinated intersectoral action in accordance with the ‘One Health’ approach*

Nowadays, there is a high degree of awareness about the appropriate use of antibiotics in livestock farming, but not so much about the non-appropriate use of antibiotics in pets and the risk this poses to public health.

This Special Issue invited papers by experts working on antibiotic resistance, in very diverse fields, showing different aspects with involvement in the One Health concept. 

The studies by Grakh et al. and Sani Ismaila et al. evaluated the causes of the inappropriate use of antibiotics in small animal clinics in areas as disparate as India, Trinidad, and Jamaica [1,2]. They show how the indiscriminate usage and overuse of antimicrobials in pets or companion animals are underlying causes of antimicrobial resistance; the reasons are incomplete courses of antibiotics, inappropriate follow-ups, the improper care of sick animals, the self-prescription of antimicrobials by owners, unavailability of antibiogram facilities, and the statement of an empirical treatment based on their experience as the main criteria for antimicrobial choice in the absence of timely results from the laboratory or the lack of use of standard antimicrobial protocols, which could be due, in some cases, to the limited availability of resources.

In the paper by Melgarejo et al., the AMR-gene presence in microorganisms recovered from urine from clinically healthy dogs is reported to highlight public health considerations in the context of a species-spanning framework [3]. From the 30 AMR genes detected, the most common AMR genes were *aph(3′)Ia*, and *ermB*, which confer resistance to aminoglycosides and MLS (macrolides, lincosamides, and streptogramins) antibiotics, respectively. These AMR genes are mainly expressed in bacterial species such as *Streptococcus*, *Staphylococcus*, and *Corynebacterium* genera. The presence of AMR genes that confer resistance to clinical important antibiotics suggests that dogs may serve as reservoirs of clinically relevant resistomes.

In a similar field, the contribution from Ahn et al. determines the effects of tetracycline at chronic subinhibitory exposure levels on human intestinal microbiota using an in vitro continuous flow bioreactor [4]. With this technique, they show that dose-dependent effects of tetracycline were observed as perturbations of *tetB* and *tetD* gene expression and changes in acetate and propionate concentrations. This contributes to knowledge on the impact of the chronic exposure of tetracycline on human intestinal microbiota.

The following articles deal with different factors that can affect the more rational and appropriate use of antibiotics and the causes that can influence their management. 

Lorenzutti et al. describe the effect of a fluoroquinolone (marbofloxacin) on *S. aureus* isolated from mastitis goat milk by different approaches as the minimum inhibitory and bactericidal concentrations (MICs and MBCs) in cation-adjusted Mueller–Hinton broth (CAMHB) and the serum and milk of goats at two inoculum sizes of 10^5^ and 10^8^ CFU/mL, respectively [5]. The authors analyzed the time kill curves (TKC) using non-linear mixed effect models in each growth medium and inoculum size, as well as the estimation of their pharmacokinetic/pharmacodynamic (PK/PD) cut-off values. The results obtained indicate that MIC values and PK/PD cut-off values to achieve clinical efficacy were highly dependent on the inoculum and growth media, suggesting that further studies are necessary to evaluate and optimize the best therapeutic strategies for treating *S. aureus* in lactating goats.

On the other hand, Nihat Ural and K. Uney studied the influence of the co-administration of fluoroquinolone (danofloxacin) with meloxicam in healthy lambs and lambs with respiratory infections [6]. The results show that co-administration with meloxicam reduces the clearance and volume of distribution, whereas AUC and Cmax are increased in infected lambs. Additionally, this co-administration can provide optimum values of ƒAUC0–24/MIC (>56 h) and ƒCmax/MIC (>8) for susceptible *M. haemolytica* isolates with an MIC90 value of 0.25 µg/mL and susceptible E. coli isolates with an MIC value of ≤0.125 µg/mL.

The study by Waxman et al. provides information about the intramuscular pharmacokinetics of enrofloxacin in black vultures (*Coragyps atratus*) to dispose the necessary knowledge to avoid extrapolation from other species and the derived risks [7]. Using previously published MIC values, the authors performed a PK/PD analysis, with cumulative fraction responses obtained after Monte Carlo simulation for AUC/MIC > 30, 50, and 125, and Cmax/MIC for *E. coli* and *Mycoplasma synoviae*. The results indicate that the doses used could be appropriate to treat infectious diseases caused by Gram-positive bacteria with MIC values lower than 1 µg/mL; however, plasma concentrations were insufficient to reach the established Gram-negative breakpoints.

Finally, in an additional line of alternative methods to improve the state of health and immunity, and therefore, the ability to respond to infections in cattle, Grossi et al. studied the effect of a nutraceutical mixture, based on live yeast, mannan-oligosaccharides, and organic selenium [8]. To this end, they carried out studies monitoring the incidence of bovine respiratory disease and other health issues, as well as the mortality rate.

In the treatment group, the occurrence of bovine respiratory disease tended to be reduced and the BHV-1 antibody production after vaccination was significantly improved, as was the bactericidal activity. Additionally, the average daily gain and final weight were significantly improved. Therefore, these results suggest that the nutraceutical mixture can support the animal’s immune systems, improving its ability to react against pathogens, as well as the feed efficiency and growth performance during the whole fattening period.
